# Assessing Falling Number Stability Increases the Genomic Prediction Ability of Pre-Harvest Sprouting Resistance in Common Winter Wheat

**DOI:** 10.3390/genes15060794

**Published:** 2024-06-17

**Authors:** Theresa Albrecht, Michael Oberforster, Lorenz Hartl, Volker Mohler

**Affiliations:** 1 Bavarian State Research Center for Agriculture, Institute for Crop Science and Plant Breeding, 85354 Freising, Germany; theresa.albrecht@lfl.bayern.de (T.A.); lorenz.hartl@lfl.bayern.de (L.H.); 2 Austrian Agency for Health and Food Safety (AGES), Institute for Sustainable Plant Production, Spargelfeldstr. 191, 1220 Vienna, Austria

**Keywords:** genomic selection, germination index, QTL, seed dormancy, *Triticum aestivum*

## Abstract

Pre-harvest sprouting (PHS) resistance is a complex trait, and many genes influencing the germination process of winter wheat have already been described. In the light of interannual climate variation, breeding for PHS resistance will remain mandatory for wheat breeders. Several tests and traits are used to assess PHS resistance, i.e., sprouting scores, germination index, and falling number (FN), but the variation of these traits is highly dependent on the weather conditions during field trials. Here, we present a method to assess falling number stability (FNS) employing an after-ripening period and the wetting of the kernels to improve trait variation and thus trait heritability. Different genome-based prediction scenarios within and across two subsequent seasons based on overall 400 breeding lines were applied to assess the predictive abilities of the different traits. Based on FNS, the genome-based prediction of the breeding values of wheat breeding material showed higher correlations across seasons (r=0.505−0.548) compared to those obtained for other traits for PHS assessment (r=0.216−0.501). By weighting PHS-associated quantitative trait loci (QTL) in the prediction model, the average predictive abilities for FNS increased from 0.585 to 0.648 within the season 2014/2015 and from 0.649 to 0.714 within the season 2015/2016. We found that markers in the *Phs-A1* region on chromosome 4A had the highest effect on the predictive abilities for FNS, confirming the influence of this QTL in wheat breeding material, whereas the dwarfing genes *Rht-B1* and *Rht-D1* and the wheat–rye translocated chromosome T1RS.1BL exhibited effects, which are well-known, on FN per se exclusively.

## 1. Introduction

Pre-harvest sprouting (PHS) describes the germination of mature seeds whilst attached to the plant. During germination, α-amylase degrades starch, and the change in starch viscosity causes a low falling number (FN), an important economic measurement for estimating the dough quality of wheat [1]. It is well known that the interdependency of weather and genotype during grain maturation affects the extent of PHS [2]. The depletion of dormancy before harvest is influenced, amongst others, by the temperature sum reached since the sowing date. In the light of forthcoming higher year-to-year variations in temperature and rainfall patterns, breeding for PHS resistant wheat cultivars will remain mandatory to minimize this risk. The whole value chain from farmers to the milling industry will benefit from PHS tolerant cultivars that show stable falling numbers also across environments that are conducive to PHS. However, without PHS-inducing conditions, a differential response of wheat cultivars in the falling number test cannot be assessed, and hence, variety registration and recommendation trials do not properly account for falling number stability (FNS). General methods for assessing PHS include the determination of falling number, germination indices, and rates of sprouting damage on threshed seeds or visible sprouting on intact ears. These tests use plant materials harvested at physiological maturity or after natural or artificial weathering in the field. While the germination indices account for germination-inducing processes and dormancy, the falling number captures several processes in the kernel including germination and enzyme activities that lead to starch degradation [3].

In previous studies, many quantitative trait loci (QTL) and genes have been identified and found to influence PHS resistance in wheat. Tai et al. [2] summarized 188 QTL from 40 studies and identified 66 meta-QTL that were distributed across all 21 chromosomes of the wheat genome, firmly documenting that PHS resistance is a complex trait. Several genes for PHS resistance have already been identified: *Viviparous* (*TaVp-1*) [4], red grain color gene *R-1* (*TaMYB10-A1* [5]; *TaMYB10-D1* [6]), DELAY OF GERMINATION1 (*TaDOG1*-like genes) [7,8], *mother of flowering time* (*TaMFT-3A* [9]; *TaPhs1* [10]), *mitogen-activated protein kinase kinase3* (*TaMKK3* = *TaMKK3-A*—the candidate gene for the *Phs1* = *Phs-A1* locus on chromosome 4A) [11,12,13], *seed dormancy* (*TaSdr-B1* [14]; *TaSdr-A1* [15]; *TaQSd1-5B* [16]), and *ABI5 binding protein* (*TaAFP-B1*) [17]. These genes were characterized to have either large or small effects on trait variation (e.g., reviewed by Vetch et al. [18]). 

In previous projects, we could confirm that PHS resistance is controlled by several large- and small-effect loci. We also identified several molecular markers associated with falling number and PHS resistance in biparental mapping populations and a diversity panel consisting of German and Austrian common winter wheat varieties and advanced breeding lines [19,20,21,22]. For complex traits under quantitative genetic control, genomic prediction using genome-wide marker data has been demonstrated useful for assessing the breeding values of selection candidates in early generations [23,24]. The recording of PHS-relevant traits is time- and cost-consuming, and a selection based on these traits of superior genotypes is only possible late in the breeding cycle. With genomic prediction, it is possible to predict the breeding values of selection candidates early in the breeding cycle based on phenotypes assessed on a small training population of advanced breeding lines that were evaluated across several environments. However, to predict the falling number stability of new selection candidates using genome-wide marker data, it is crucial to properly assess the trait variation that is intrinsic to the training population, because this will influence the predictive abilities across years. Therefore, we used a procedure for reducing dormancy consisting in subjecting the kernels to an after-ripening period before assessing the falling number.

In this study, we applied genomic prediction in advanced common winter wheat material derived from German and Austrian breeding programs. The objectives of this study were to (1) present a method to assess falling number stability under controlled conditions; (2) apply genomic prediction models across years, additionally considering PHS resistance QTL to improve the predictive abilities.

## 2. Materials and Methods

**Figure 1:**
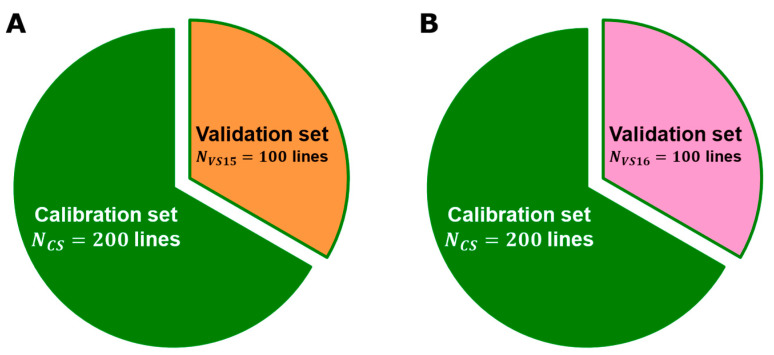
Overview of the data set split into a calibration set (CS) and two validation sets (VS15 and VS16) analyzed in the (**A**) season 2014/2015 and (**B**) season 2015/2016.

The complete data set included 400 breeding lines that were derived from six companies/programs (Saatzucht Josef Breun GbR, Herzogenaurach, DE: 80 lines, Saatzucht Edelhof, Zwettl, AT: 80 lines, Limagrain GmbH, Rosenthal, DE: 80 lines, Secobra Saatzucht GmbH, Moosburg an der Isar, DE: 94 lines, Lantmännen SW Seed GmbH, Hadmersleben, DE: 20 lines, and Bavarian State Research Center for Agriculture: 46 lines). The material represented F_6_ lines of two subsequent seasons that were not selected for PHS resistance but were selected according to the breeder’s specific selection program for winter wheat. In the season 2014/2015, 300 lines of the corresponding breeding programs were analyzed. To establish a calibration set, 200 lines were selected equally across the breeding pools from the previous season, and analyzed together with 100 new selection candidates in the season 2015/2016. Therefore, the data set of 400 lines could be divided into a calibration set (CS) evaluated in two seasons, consisting of 200 lines, and two validation sets (VS15 and VS16), composed of 100 lines each, evaluated in the respective season (Figure 1). 

### 2.1. Field Trials

In each season, 300 breeding lines were phenotyped at five locations (Rosenthal (52.30092 N, 10.16959 E), Feldkirchen (48.48019 N, 11.90780 E), and Herzogenaurach (49.56910 N, 10.87840 E) in Germany; Zwettl (48.6075 N, 15.22458 E) and Fuchsenbigl (48.20021 N, 16.74489 E) in Austria). Field trials were performed using an unreplicated augmented design with ten blocks and three varieties as controls with different responses to PHS-inducing conditions (Julius, high PHS resistance; JB Asano, medium PHS resistance; Colonia, low PHS resistance) repeated in each block. The 300 breeding lines were randomly assigned as entries across blocks.

### 2.2. Phenotyping

Five traits were used to assess pre-harvest sprouting: germination index (GI), sprouting of intact ears under controlled conditions (LS, lab sprouting), falling number per se (FN1) and after either natural or artificial rain (FN2), and falling number stability (FNS).

For the germination test, 100 seeds from each plot of each genotype (repeated four times) were uniformly spread between filter papers in germination boxes saturated with 4 mL of tap water and incubated at room temperature. The seeds with an exposed tip of the coleoptile were defined as “germinated”, and the germinated seeds were counted and removed over 6 days. GI was computed with maximum weight given to the seeds that germinated first and less weight to those that germinated later, following the formula proposed by Rasul et al. [25].

For the LS tests, 10 spikes from each genotype were randomly assigned to a rain simulation chamber (99–100% humidity, 20 °C in the dark) in Austria. As a second method in Germany, wetted spikes were incubated in perforated plastic trays under humid conditions according to Albrecht et al. [20]. After one week, the sprouting scores based on an arbitrary scale from 1, indicating no sprouting, to 9, indicating severe sprouting, were determined.

The effects of α-amylase activity were measured on the falling number instrument FN 1700 (Perten Instruments, Hägersten, SE), according to the standard method no. 107/1 of the International Association for Cereal Science and Technology. The falling numbers in German locations were assessed at physiological maturity of the kernels (FN1) and after the plants had experienced a period of natural weathering (FN2). In Austria, without exception, all harvests were delayed, after either artificial (Fuchsenbigl) or natural rain (Zwettl). For an increased differentiation of the dormancy levels within the breeding material under weather conditions that were not conducive to PHS, 33 g of grain samples was stored in the fridge to conserve dormancy until testing. The procedure to determine the FNS of the samples consisted of an after-ripening period at room temperature, the soaking of the kernels, and the determination of the falling number after drying the kernels [26]. The optimal length of the after-ripening period for each location was determined based on the control cultivars, which were stored for 0, 2, and 4 weeks at room temperature. The after-ripening period was followed by the coating of the samples with 16.5 mL of water in Petri dishes (150 mm diameter) for 24 h to induce the germination process. After drying the wet kernels for 18 h at 55 °C, the falling numbers were determined as described before.

In addition to the PHS traits, plant height (PH in cm, measured from the ground level to the top of the canopy after flowering) and days to heading (HDs, recorded as the number of days from May 1st until the emergence of 50% of the spikes in a plot) were assessed in six environments for evaluating the quality of the field trials.

### 2.3. Genotyping

The set of 400 lines was genotyped with the Illumina^®^ 15k Infinium iSelect BeadChip by TraitGenetics GmbH. Quality control and analysis of the marker data were performed in R with the “synbreed” package [27]. The final marker data were recoded according to the copies of the minor allele observed for each marker, with 0 indicating homozygosity for the major allele, 1 heterozygosity, and 2 homozygosity for the minor allele. Three lines were discarded for further analysis due to missing values (>20%) or heterogeneity. Principal component analysis was applied to all remaining 397 lines for structural analysis of the data set.

Seventeen specific markers which targeted ten candidate genes or QTL were developed based on publicly available sequence resources and analyzed in-house on a Biomark X platform (Standard BioTools™, South San Francisco, US). All markers were found in previous experiments to influence PHS-related traits in German winter wheat breeding material (Table 1). In addition, three markers were analyzed that captured the wheat–rye translocation T1RS.1BL and the dwarfing genes *Rht-B1* (*Rht1*) and *Rht-D1* (*Rht2*), known to influence the FN per se.

### 2.4. Statistical Analysis

Trait repeatability was assessed for each single environment using a model with random effects for entries and blocks. Trait heritability and adjusted means were calculated across all locations based on raw phenotypic values for each season separately, using a model with block effects nested in locations, genotype effects, and genotype-by-location interaction.

The genome-based breeding values (GEBVs) were predicted using best linear unbiased prediction (GBLUP) based on adjusted means as vector of phenotypes **y** according to the following model:

y=Xβ+Zt+e

where **β** is a vector of fixed effects, and **X** is a design matrix assigning fixed effects to the phenotypes. In model 1, the fixed effects included only the population means, whereas in model 2, the fixed effects included the effects for the 17 specific markers and **X** assigned marker genotypes to phenotypes. The vector of breeding values **t** follows a normal distribution with ${t\sim N(0,A\sigma }_{t}^{2}),$ where **A** is the marker-based relationship matrix according to VanRaden [31], and $\sigma_{t}^{2}$ is the genetic variance. The design matrix **Z** assigns breeding values to phenotypes. The vector of residual effects **e** follows a normal distribution with ${e\sim N(0,I\sigma }_{e}^{2})$, where **I** is an identity matrix, and $\sigma_{e}^{2}$ is the residual variance. Two additional variants of model 2 were calculated that weighted either the major PHS resistance locus *Phs-A1* or a combination of the wheat–rye translocation T1RS.1BL and the dwarfing genes *Rht-B1* (*Rht1*) and *Rht-D1* (*Rht2*).

### 2.5. Cross-Validation

For a comparison of the prediction models, different cross-validation (CV) schemes were applied. In each season, fivefold cross-validation with ten replications was used to assess the predictive abilities of both models, taking the different breeding programs into account [24,27]. Here, the lines from each breeding program were randomly divided into five sets of equal size, and four sets formed the estimation set (ES) to predict the lines in the 5th set, i.e., the test set (TS). The prediction was performed five times, so that every subset was used as a test set. This sampling strategy considered that the ES and the TS always included lines from each breeding program and that each line was used once in the TS. The sampling of the subsets was repeated ten times, so that overall, 50 predictions were performed. The predictive abilities within each season were calculated as correlations between genome-based predicted and observed breeding values (OBVs) in each test set (*r*($y_{TS},{\hat{g}}_{TS}$)), where the predicted values in the test set were calculated as ${\hat{g}}_{TS}=X_{TS}\hat{\beta }+Z_{TS}\hat{t}$. Differences between the predictive abilities of models 1 and 2 were examined with a Student’s paired *t*-test in R.

For the validation across seasons, the lines evaluated in one season were used to predict the lines from the other season using different prediction scenarios and model 2. Here, the prediction panel used for calibrating the model included all lines from one season ($N_{ES}=298$) or the lines evaluated in both seasons ($N_{ES}=199$). The predictive abilities were calculated either based on the lines evaluated in both seasons ($N_{TS}=199$) or based on the lines evaluated in only one season ($N_{TS}=99$). All statistical analyses were performed in R version 3.4.0 [32] with the packages “lme4” [33], “lsmeans” [34], and “synbreed” [27].

## 3. Results

### 3.1. Phenotypic Analysis

The quality of the field trials was high, as indicated by the high heritability for PH ($h_{PH}^{2}=0.95$ in 2015 and $h_{PH}^{2}=0.88$ in 2016) and HDs ($h_{HD}^{2}=0.86$ in 2015 and $h_{HD}^{2}=0.92$ in 2016). Except for Rosenthal 2015, where no sprouting was observed after the artificial wetting of intact ears, sufficient trait variation was observed at most locations (Figures S1 and S2). In the environment Feldkirchen 2016, the field trial was harvested late, and therefore, the falling numbers were assigned to FN2 instead of FN1. The level of dormancy in the samples was higher in the season 2014/2015 than in the season 2015/2016. Thus, for samples harvested in 2015, the after-ripening period for the FNS test was up to four weeks, while for most locations in 2016, no after-ripening was necessary to assess FNS. The trait repeatability for single locations ranged from 0.24 to 0.95 (Table 2). In general, repeatability was higher than 0.5, except for Feldkirchen 2015 (${rep}_{GI}^{2}=0.39$) and Rosenthal 2016 (${rep}_{FN2}^{2}=0.24$, ${rep}_{FNS}^{2}=0.35$). Trait heritability was consequently high and ranged from 0.58 for FN1 in 2015 to 0.88 for FNS in 2016.

### 3.2. Genotypic Analysis

**Figure 2:**
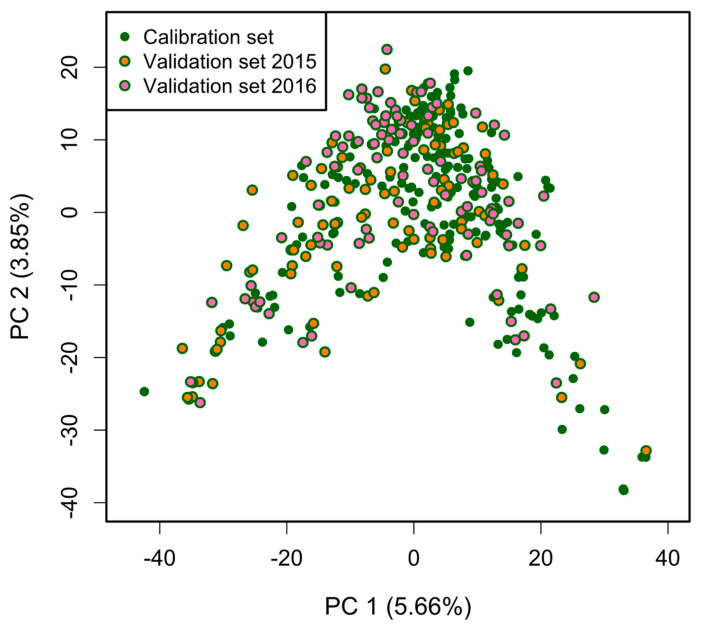
Scatterplot of the first two principal components (PCs) based on 297 wheat lines (colored according to the corresponding calibration and validation sets) and 6244 SNP markers.

The genotyping resulted in 13,006 SNPs, and after quality control, 6244 SNPs with minor allele frequency (MAF) >0.01, missing values <5%, and no duplicates in the data set remained for further analysis. The average MAF across all SNPs was 0.233. The first two principal components explained only 5.66% and 3.85% of the genetic variance in the data set, and CS, VS15, and VS16 were equally spread across the complete data set (Figure 2). The German and Austrian breeding pools separated along the first principal component, with a large overlap in the center of the first two principal components (Figure S3) indicating no substantial population structure across the breeding pools. The MAF for the 17 Biomark X platform markers ranged from 0.037 for BS00009060_51 to 0.483 for wPt-3790, with an average MAF of 0.252 across the breeding lines. The markers representing T1RS.1BL, *Rht-B1*, and *Rht-D1* had a MAF of 0.414, 0.217, and 0.486, respectively. 

### 3.3. Predictive Abilities for the PHS Traits within and across Seasons

**Figure 3:**
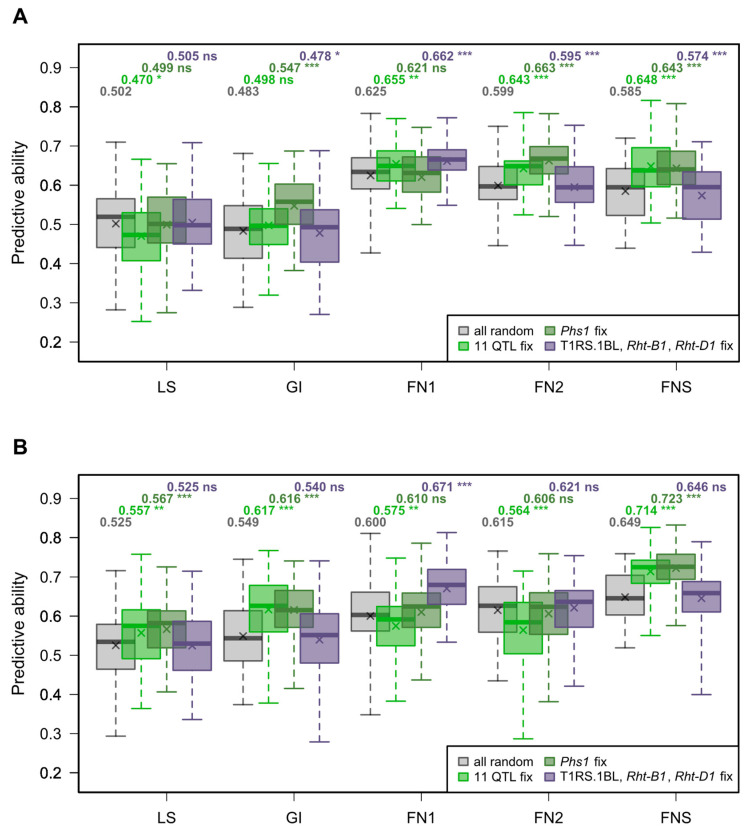
Boxplots of the predictive abilities based on 10 × 5-fold cross-validation using model 1 (grey) and model 2 with different sets of markers for (light green) 11 QTL, (dark green) *Phs1*, and (violet) *Rht-B1*, *Rht-D1*, and the translocation T1RS.1BL as fixed effects within the seasons (**A**) 2014/2015 and (**B**) 2015/2016 using the complete data set (CS + VS) for the traits lab sprouting (LS), germination index (GI), falling number per se (FN1), falling number after rain (FN2), and falling number stability (FNS). The average predictive abilities are presented above the corresponding boxplots, with significance levels based on a Student’s paired *t*-test comparing model 1 with model 2. ns: p≥0.05; *: p<0.05; **: p<0.01; ***: p<0.001.

Figure 3 shows the predictive abilities for 50 cross-validation runs of model 1 and 2 in both seasons. The average predictive abilities for model 1 ranged from 0.483 to 0.625 in the season 2014/2015 and from 0.525 to 0.649 in the season 2015/2016. With the fitting of 17 markers linked to PHS resistance QTL in model 2, the average predictive abilities increased for most of the traits and ranged from 0.470 to 0.655 in the season 2014/2015 and from 0.557 to 0.714 in the season 2015/2016. Weighting only *Phs-A1*, represented by three markers on chromosome 4A (Table 1), in model 2 significantly increased the predictive abilities compared to those of model 1 for GI and FNS in both seasons. The highest predictive abilities were observed for FNS and model 2 considering ten QTL or only *Phs-A1* in both seasons. A significant increase (p<0.001) in the average predictive abilities was observed for FN1 when a single marker each for T1RS.1BL, *Rht-B1*, and *Rht-D1* was used as a fixed effect in the model.

**Figure 4:**
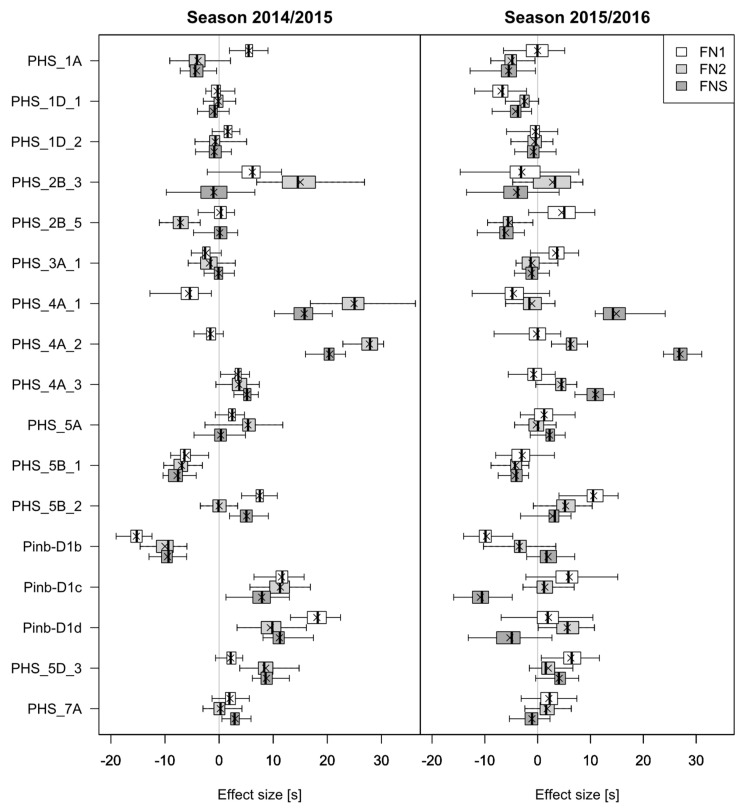
Boxplots of the estimated effects for 17 PHS markers with model 2 using 10 × 5-fold cross-validation for trait falling number per se (FN1), falling number after rain (FN2), and falling number stability (FNS) in the seasons 2014/2015 and 2015/2016.

Figure 4 illustrates the estimated fixed effects of the markers linked to PHS resistance QTL in model 2 for the traits FN1, FN2, and FNS of all 50 cross-validation runs in both seasons. The highest effects were observed for the traits FN2 and FNS for the markers Kukri_c12563_52 and BS00072025_51 linked to *Phs-A1* on chromosome 4A. For FNS, this effect was consistent across both seasons, whereas for FN2, the effect size decreased in the season 2015/2016. In Figure S4, the effect of the haplotypes derived from these two markers for *Phs-A1* on FNS is illustrated. In both seasons, the genotypes carrying the largest haplotype class (G:G/T:T) had significantly (p<0.05) lower falling numbers compared to the other two haplotypes. The same effect could be observed for FN2 (Figure S5), but in the season 2015/2016, the average FNs of all haplotype classes were higher compared to those in the season 2014/2015, and the differentiation of the haplotype classes was reduced. A decrease in effect size for FN2 could be also observed for the marker BS00009060_51 on chromosome 2B (Figure 4). For FN1, large effects could be observed for the markers targeting three different *Pinb-D1* gene variants in the season 2014/2015.

In Table 3, the correlations between GEBVs and OBVs across the examined seasons based on different estimation and test sets are presented. The correlations ranged from 0.216 for GI, using the data from the season 2015/2016 to predict the statistically independent VS15, to 0.763 for FNS, when the CS16 was predicted based on the data from the season 2014/2015. The correlations between GEBVs and OBVs of the independent VSs were generally lower than those predicting the same material in the CS across the seasons. The correlations between OBVs of the CS in both seasons were also lower than the correlations between GEBVs within the CS, estimated in each season. Here, the correlation between GEBVs across the seasons was highest for GI (r=0.817).

## 4. Discussion

The success of genomic prediction in plant breeding depends on the accuracy achieved with a prediction model. PHS resistance is a complex trait, and many genes/loci controlling PHS have already been detected [2,35]. Therefore, MAS with a reduced marker panel is not useful, and mixed models taking large marker panels into account have been suggested [36,37]. Different mixed models using BLUP, Bayesian, or machine learning approaches have been developed [38,39,40,41]. However, so far none of these models was able to outperform GBLUP in wheat breeding [35,42]. Hence, we chose GBLUP as the fastest computational method.

In our data set built-up on the information from breeding populations, trait heritability for GI and LS was lower compared to the heritability achieved in a diverse winter wheat panel [20]. Michel et al. [43] suggested to select the best of the trials to compute GEBVs to improve heritability and, hence, prediction accuracies. However, when the number of trait assessments is limited, e.g., for baking tests of wheat, which are time-consuming and cost-intensive to determine, or trait assessment is highly dependent on optimal weather conditions such as it is for PHS resistance, a pre-selection of the trials will not be feasible. Therefore, our study aimed at increasing the predictive accuracies by improving heritability through a better trait assessment independent of the weather conditions. In a recent study [44], PHS was assessed with the falling numbers after artificial rain and delayed harvest (FN2). Therefore, we compared FN2 with our in-house-established falling number stability test (FNS). Although, in our study, heritability for FN2 was high, we could achieve more consistent trait variation and even a higher heritability for FNS across locations and seasons (Table 2), which was equivalent to the heritability for LS and GI achieved with a diverse wheat panel [20]. The highest average predictive abilities were observed for FNS and model 2 within the season 2015/2016. For predicting GEBVs across the examined seasons, the correlations for FNS achieved in each scenario were higher compared to those for FN2 (Table 3). Thus, the predictive abilities increased by 22% on average across all prediction and correlation scenarios. In comparison to LS and GI, the predictive abilities for FNS improved by an average of 27% and 26%, respectively. With respect to FN1, the average increase was only of 7%. However, in our study, FN1 represented the FN per se under non-PHS-inducing conditions, and the predictive abilities for FN1 might not be transferable to seasons with PHS. Therefore, we recommend the application of an after-ripening period and the wetting of the kernels to assess FNS for improving the genomic prediction of PHS resistance in breeding material.

As demonstrated by simulation, genomic prediction in breeding populations benefits from taking major QTL into account for heritable traits ($h^{2}>0.8$) and QTL explaining together more than 50% of the genetic variation of the trait [36]. In this simulation study, no gain was observed when modelling more than one major QTL if the breeding populations were small. As PHS resistance is a complex trait controlled by many genes with moderate to small effects, we chose 17 markers associated with 11 QTL detected in previous analyses to determine their influence on five different PHS-related traits. Two of these QTL were *TaMFT-3A* and *Phs-A1*, which were identified to have a large effect on PHS resistance. We found the predictive abilities to be similar or increased by incorporating 11 QTL as fixed effects in the model (Figure 3). However, the effects of these QTL were not consistent across years for the traits LS, GI, FN1, and FN2. Only for FNS, the predictive abilities within each season increased consistently. The same observations were made for the effects estimated with cross-validation for each single marker in model 2, which were not consistent across the seasons, except for FNS. The largest effects were observed for *Phs-A1* on chromosome 4A for FN2 and FNS. This became even better visible in the haplotype analysis of *Phs-A1* for FNS, where the genotypes were based on the two SNP markers Kukri_c12563_52 and BS00072025_51 (Figure S4). In both seasons, the haplotypes A:A/T:T and G:G/C:C were associated with the highest FNS values, whereas G:G/T:T for Kukri_c12563_52/BS00072025_51 showed the lowest values for FNS. Similar effects were observed for FN2, but the average FN in each haplotype class changed across the seasons (Figure S5), and the effects for these two markers diminished in the prediction model due to a lower number of field trials with PHS-conducive conditions in the season 2015/2016 (Figure 4). This is in accordance with previous studies where *Phs-A1* appeared as one of the major QTL explaining up to 50% of the phenotypic variation for PHS-related traits [44,45]. Our results from weighting *Phs-A1* as a fixed effect in the prediction model clearly underline the importance of this locus. Moore et al. [37] identified *TaMFT-3A* by association mapping and demonstrated that modelling this QTL as a fixed effect increased the predictive abilities for GI. For the marker CAP12_c1860_280 located in the *TaMFT* region on chromosome 3A, only small effects were observed with model 2. Therefore, we assume that our marker used was not clearly indicative for *TaMFT-3A*. For *Pinb-D1*, only the mutant allele *Pinb-D1b* had a consistent effect on FN1 across both seasons (Figure 4). This is in accordance with a recent publication by Muqaddasi et al. [43], where only *Pinb-D1b* influenced FN per se compared to the wild-type allele *Pinb-D1a*. However, it appears that *Pinb-D1* is generally not associated with FN per se [28].

These results show that mainly *Phs-A1* contributes to PHS resistance in breeding populations, while *Phs-A1* does not show an effect on FN per se. For FN1, a model with three markers for the dwarfing genes *Rht-B1* and *Rht-D1* and the wheat–rye translocated chromosome T1RS.1BL led to higher predictive abilities (Figure 3), confirming that FN per se is controlled, inter alia, by these major wheat breeding signatures. Therefore, we suggest taking only *Phs-A1* as a fixed effect in the model for predicting PHS resistance. Furthermore, in small breeding populations, it might be beneficial to screen for PHS-resistant lines based on the haplotypes of the markers Kukri_c12563_52 and BS00072025_51, representing *Phs-A1*.

## 5. Conclusions

In this study, we showed that the prediction of PHS resistance in new breeding material is feasible for its implementation into breeding programs. The predictive abilities could be increased by weighting known QTL as fixed effects in the genomic prediction model [36,46]. Here, the QTL *Phs-A1* mainly contributed to PHS resistance, and the candidate gene *TaMKK3* underlying *Phs-A1* has already been described. However, as presented in our results, most of the breeding lines carried haplotypes for *Phs-A1* with reduced PHS resistance, which makes *TaMKK3* interesting for the application of new breeding technologies such as CRISPR/Cas9 to improve PHS resistance in wheat breeding material, as already demonstrated for *Tamyb10* [47]. We could demonstrate that improved trait differentiation by a controlled after-ripening period before assessing the FN increased the predictive abilities for PHS resistance. Furthermore, the effects of haplotypes and QTL modelled for prediction were more consistent for FNS across the seasons compared to those obtained with standard procedures to assess PHS resistance. In combination with a fast and cheap method to measure the FN using spectrometers [48], the FNS test can improve the selection of PHS-resistant wheat breeding lines.

## Figures and Tables

**Table 1:** Markers in PHS- and FN-QTL regions from previous projects. Markers targeting similar genome positions are assumed to represent the same QTL.

QTL Marker	Identifier	TCAP Code	Chromosome	Position (bp) ^a^	Reference
*PHS_1A*	wsnp_Ex_c14866_22995097	IWA1952	1A	**344966381**	[20]
*PHS_1D_1*	wPt-3790		1D	**20655831**	[20]
*PHS_1D_2*	Kukri_c12758_2101	IWB40888	1D	**412771500**	[21]
*PHS_2B_3*	BS00009060_51		2B	747821525	[21]
*PHS_2B_5*	wsnp_Ex_rep_c103064_88104690	IWA5081	2B	**759573938**	[21]
*PHS_3A_1*	CAP12_c1860_280	IWB13256	3A	8687027	*TaMFT-3A*; [21]
*PHS_4A_1*	Kukri_c12563_52	IWB40846	4A	**604639304**	*Phs-A1*; [21]
*PHS_4A_2*	BS00072025_51		4A	**604570739**	*Phs-A1*; [21]
*PHS_4A_3*	RAC875_c21369_425	IWA7058	4A	**605271799**	*Phs-A1*; [21]
*PHS_5A*	wsnp_Ex_c18941_27840714	IWA2363	5A	**548346335**	[21]
*PHS_5B_1*	wsnp_Ex_rep_c108314_91592072	IWA5166	5B	**339326692**	[20]
*PHS_5B_2*	wsnp_Ku_c8270_14083963	IWA7318	5B	**324715026**	[19]
*Pinb-D1b*	Pinb-D1b		5D	**3622739**	[28]
*Pinb-D1c*	Pinb-D1c		5D	**3622739**	[28]
*Pinb-D1d*	Pinb-D1d		5D	**3622739**	[28]
*PHS_5D_3*	Kukri_rep_c73094_348	IWB50247	5D	**281399038**	[21]
*PHS_7A*	wsnp_Ku_c3929_7189422	IWA7005	7A	**737404987**	[19]

^a^ Position based on RefSeq v1.0 [29] and RefSeq v2.1 (in bold) [30].

**Table 2:** Repeatability in each environment and heritability for the traits lab sprouting (LS), germination index (GI), falling number per se (FN1), falling number after rain (FN2), and falling number stability (FNS) in the seasons 2014/2015 and 2015/2016.

Environment	Repeatability *rep²*				
	LS	GI	FN1	FN2	FNS
Season 2014/2015					
Rosenthal	-	0.747	0.605	0.859	0.816
Feldkirchen	0.690	0.386	0.676	0.712	0.757
Herzogenaurach	0.547	0.769	0.652	0.796	0.725
Zwettl	0.741	0.779	-	0.878	0.758
Fuchsenbigl	0.700	0.547	-	0.743	0.611
Heritability *h²*	0.671	0.684	0.656	0.764	0.848
Season 2015/2016					
Rosenthal	0.573	0.827	0.596	0.235	0.349
Feldkirchen	0.668	0.833	-	0.748	0.924
Herzogenaurach	0.843	0.845	0.889	0.661	0.764
Zwettl	0.799	0.847	-	0.879	0.954
Fuchsenbigl	0.859	0.833	-	0.798	0.914
Heritability *h²*	0.643	0.812	0.584	0.782	0.876

**Table 3:** Prediction and correlation scenarios across the examined seasons based on different estimation (ES) and test sets (TS) for the traits lab sprouting (LS), germination index (GI), falling number per se (FN1), falling number after rain (FN2), and falling number stability (FNS). The upper part of the table shows the correlation between GEBVs and OBVs in the TS based on a prediction with model 2. The lower part of the table shows the correlation between the OBVs of the CS in each season or between the GEBVs of the CS estimated in each season.

**Prediction across Seasons**				**LS**	**GI**	**FN1**	**FN2**	**FNS**
**ES**	**TS**	***N_ES_***	***N_TS_***					
CS15 + VS15	CS16	298	199	0.615	0.747	0.732	0.558	0.763
CS16 + VS16	CS15	298	199	0.548	0.684	0.739	0.650	0.732
CS15 + VS15	VS16	298	99	0.467	0.479	0.414	0.501	0.548
CS16 + VS16	VS15	298	99	0.371	0.216	0.500	0.393	0.505
**Correlation between seasons**								
OBV_CS15	OBV_CS16	199	199	0.579	0.730	0.705	0.613	0.751
GEBV_CS15	GEBV_CS16	199	199	0.630	0.817	0.796	0.642	0.778

## Data Availability

Data are unavailable due to privacy.

## Supplementary Materials

The following supporting information can be downloaded at: https://www.mdpi.com/article/10.3390/genes15060794/s1, Figure S1: Density distribution of block-adjusted observed values for the traits (A) lab sprouting (LS), (B) germination index (GI), (C) falling number per se (FN1), (D) falling number after rain (FN2), and (E) falling number stability (FNS) of each single environment in the season 2014/2015; Figure S2: Density distribution of block-adjusted observed values for the traits (A) lab sprouting (LS), (B) germination index (GI), (C) falling number per se (FN1), (D) falling number after rain (FN2), and (E) falling number stability (FNS) of each single environment in the season 2015/2016; Figure S3: Scatterplot of the first two principal components (PCs) based on 297 wheat lines (colored according to the breeding pools) and 6244 SNP markers. Figure S4: Effect of the haplotypes of the two markers Kukri_c12563_52 and BS00072025_51 in the Phs1 region on the observed values for FNS in the seasons (A) 2014/2015 and (B) 2015/2016. Different letters above the boxplots indicate significant differences according to a Tukey’s HSD test (p=0.05), and the mean FN2 is shown below the boxplots; Figure S5: Effect of the haplotypes of the wo markers Kukri_c12563_52 and BS00072025_51 in the Phs1 region on the observed values for FN2 in the seasons (A) 2014/2015 and (B) 2015/2016. Different letters above the boxplots indicate significant differences according to a Tukey’s HSD test (p=0.05), and the mean FNS is shown below the boxplots.
