# Supplementary material for: Assessing Falling Number Stability Increases the Genomic Prediction Ability of Pre-Harvest Sprouting Resistance in Common Winter Wheat
Source: Genes (Basel). 2024 Jun 17;15(6):794. doi: 10.3390/genes15060794 (PMC11202678; doi:10.3390/genes15060794)
Supplement: Supplementary file 1 [file genes-15-00794-s001.zip › genes-3055473-supplementary.pdf]

Supplementary Material

Assessing falling number stability increases genomic prediction ability of pre-harvest sprouting resistance in common winter wheat

Theresa Albrecht [1], Michael Oberforster [2,#], Lorenz Hartl[1] and Volker Mohler [1,*]

1 Bavarian State Research Center for Agriculture, Institute for Crop Science and Plant Breeding, 85354 Freising, Germany
2 Austrian Agency for Health and Food Safety (AGES), Institute for Sustainable Plant Production, Spargelfeldstr. 191, 1220 Vienna, Austria
In memoriam
* Correspondence: Volker.Mohler@lfl.bayern.de

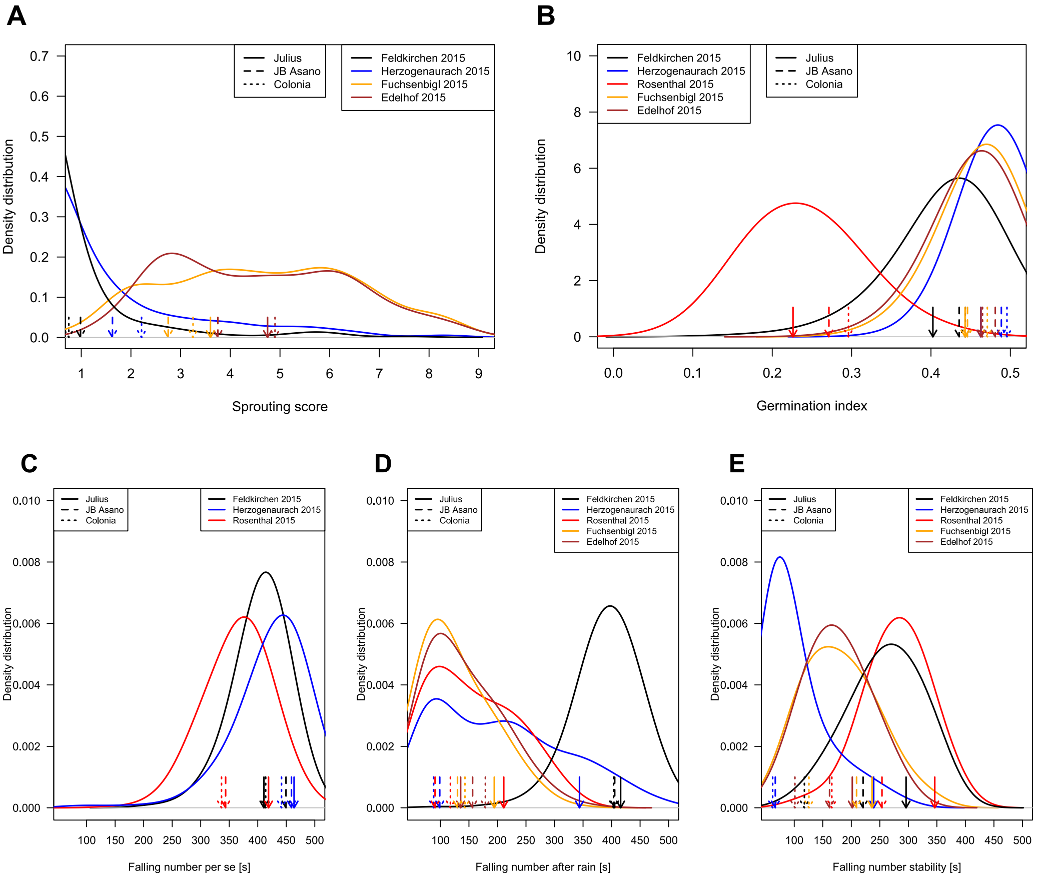

Figure S1. Density distribution of block adjusted observed values for the traits (A) lab sprouting (LS), (B) germination index (GI), (C) falling number per se (FN1), (D) falling number after rain (FN2), and (E) falling number stability (FNS) of each single environment in season 2014/2015.

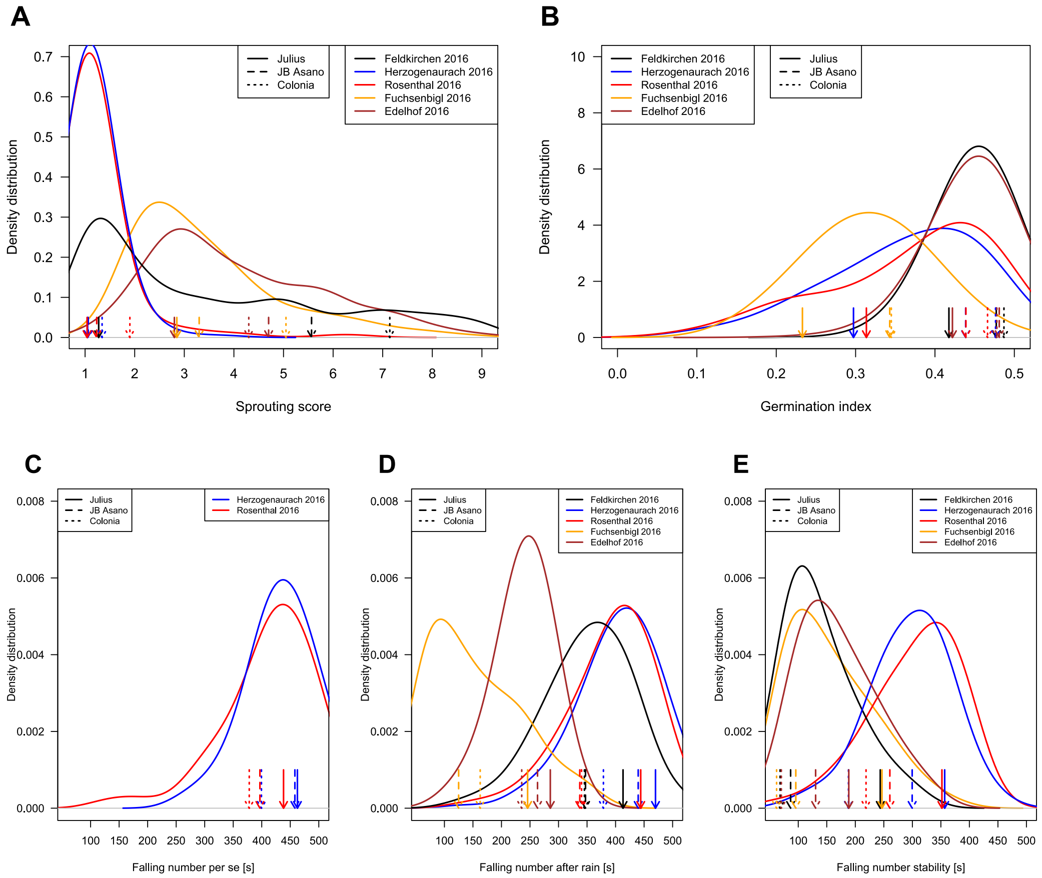

Figure S2. Density distribution of block adjusted observed values for the traits (A) lab sprouting (LS), (B) germination index (GI), (C) falling number per se (FN1), (D) falling number after rain (FN2), and (E) falling number stability (FNS) of each single environment in season 2015/2016.

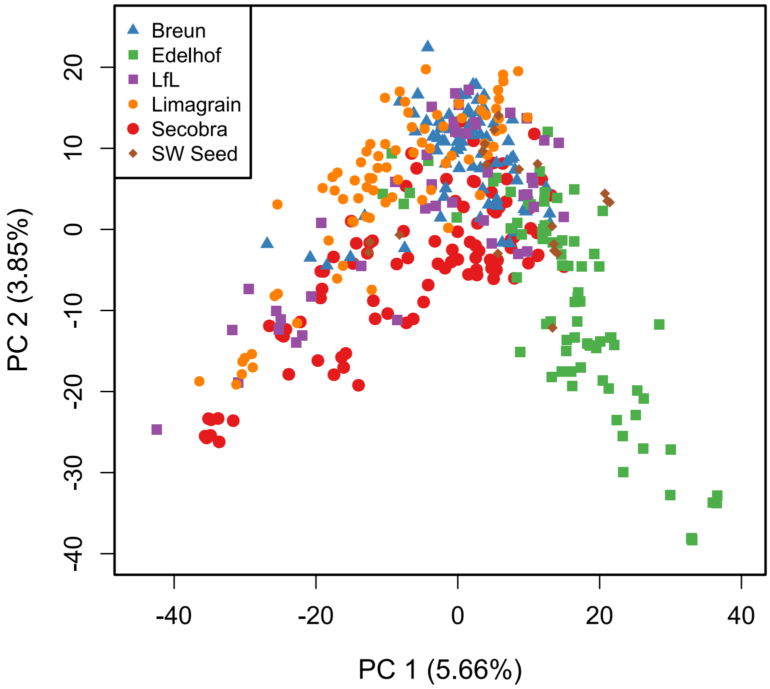

Figure S3 Scatterplot of the first two principal components (PC) based on 297 wheat lines (colored according to breeding pools) and 6244 SNP markers.

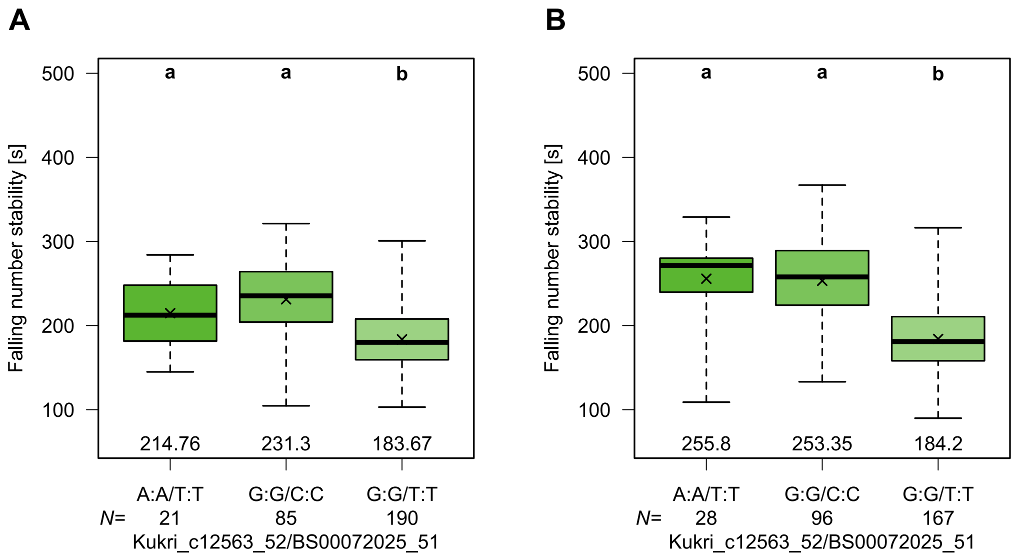

Figure S4 Effect of haplotypes of two markers Kukri_c12563_52 and BS00072025_51 in the *Phs1* region on observed values for FNS in season (A) 2014/2015 and (B) 2015/2016. Different letters above the boxplots indicate significant differences according to a Tukey's HSD test (p = 0.05) and mean FNS is shown below boxplots.

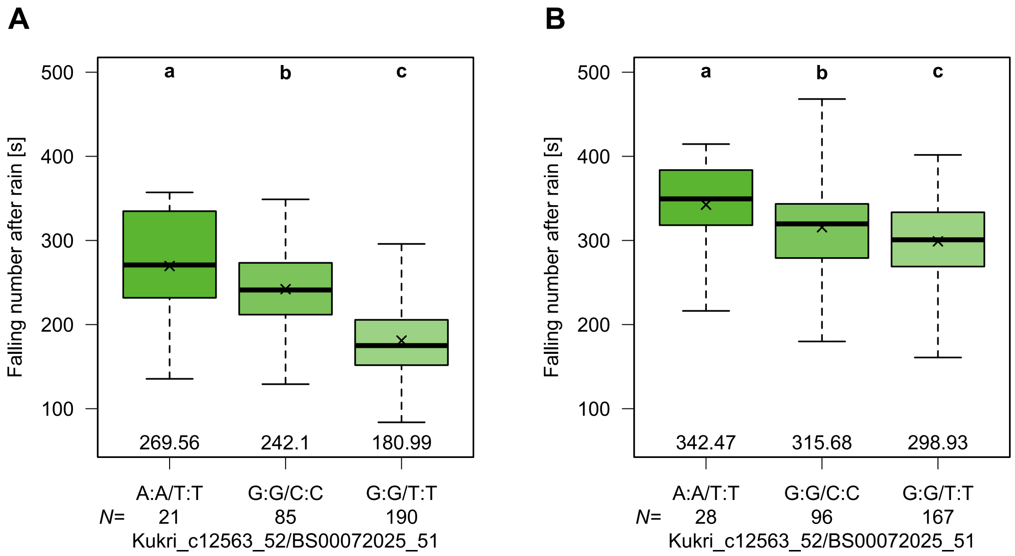

Figure S5 Effect of haplotypes of two markers Kukri_c12563_52 and BS00072025_51 in the *Phs1* region on observed values for FN2 in season (A) 2014/2015 and (B) 2015/2016. Different letters above the boxplots indicate significant differences according to a Tukey's HSD test (p = 0.05) and mean FN2 is shown below boxplots.
